# Omission of axillary lymph node dissection in ultrasound-detectable axillary metastases in primary breast cancer treated by upfront surgery: prospective randomized SENOMAC-ULTRA non-inferiority trial

**DOI:** 10.1093/bjsopen/zrag083

**Published:** 2026-08-04

**Authors:** Jana de Boniface, Tuomo Meretoja, Lisa Rydén, Malin Sund, Per Karlsson, Chiun-Sheng Huang, Hee Jeong Kim, Jai Min Ryu, Stephen Nash, Birgitte Offersen, Henrik Dahl Nissen, Elinore Wieslander, Nadia Maggi, Linda Swedin, Sung-Hsin Kuo, Trine Tramm, Karin Dembrower, Kaori Terata, Ikuno Nishibuchi, Miguel Ángel Luna Tomás, Raquel Ciérvide, Aoife Lowery, Helle Kristine Skjerven, Bent Ejlertsen, Antonios Valachis, Robert Szulkin, Tanja Spanic, Tadahiko Shien, Tove Filtenborg Tvedskov, Sara Alkner

**Affiliations:** Department of Medical Epidemiology and Biostatistics, Karolinska Institutet, Stockholm, Sweden; Department of Surgery, Capio St. Göran’s Hospital, Stockholm, Sweden; Division of Breast Surgery, Comprehensive Cancer Center, Helsinki University Hospital and University of Helsinki, Helsinki, Finland; Institute of Clinical Sciences Lund, Division of Surgery, Lund University, Lund, Sweden; Department of Surgery, Skåne University Hospital, Malmö, Sweden; Division of Breast Surgery, Comprehensive Cancer Center, Helsinki University Hospital and University of Helsinki, Helsinki, Finland; Department of Diagnostics and Intervention/Surgery, Umeå University, Umeå, Sweden; Department of Oncology, Institute of Clinical Sciences Sahlgrenska Academy, University of Gothenburg, Gothenburg, Sweden; Sahlgrenska University Hospital, Sahlgrenska Comprehensive Cancer Center, Gothenburg, Sweden; National Taiwan University Hospital, National Taiwan University College of Medicine, Taipei, Taiwan; Division of Breast Surgery, Department of Surgery, College of Medicine, University of Ulsan, Asan Medical Center, Seoul, Korea; Division of Breast Surgery, Department of Surgery, Sungkyunkwan University School of Medicine, Samsung Medical Center, Seoul, Korea; Department of Medical Epidemiology and Biostatistics, Karolinska Institutet, Stockholm, Sweden; Department of Oncology, Aarhus University Hospital, Aarhus University, Aarhus, Denmark; Department of Experimental Clinical Oncology, Danish Center for Particle Therapy, Aarhus, Denmark; Department of Oncology, Vejle Hospital, University Hospital of Southern Denmark, Vejle, Denmark; Department of Hematology, Oncology and Radiation Physics, Skåne University Hospital, Lund, Sweden; Department of Medical Epidemiology and Biostatistics, Karolinska Institutet, Stockholm, Sweden; Department of Medical Epidemiology and Biostatistics, Karolinska Institutet, Stockholm, Sweden; Graduate Institute of Oncology, National Taiwan University College of Medicine, Taipei, Taiwan; Division of Radiation Oncology, Department of Oncology, National Taiwan University Hospital, Taipei, Taiwan; Department of Pathology, Aarhus University Hospital, Aarhus, Denmark; Department of Clinical Medicine, Aarhus University, Aarhus, Denmark; Department of Medical Epidemiology and Biostatistics, Karolinska Institutet, Stockholm, Sweden; Breast Imaging Unit, Department of Radiology, Capio St. Göran’s Hospital, Stockholm, Sweden; Department of Breast and Endocrine Surgery, Akita University, Akita, Japan; Department of Radiation Oncology, Hiroshima University, Hiroshima, Japan; Department of Gynaecology, Hospital Universitario Germans Trias i Pujol, Barcelona, Spain; Department of Radiation Oncology, Hospital Universitario HM Sanchinarro, Madrid, Spain; Discipline of Surgery, University of Galway and Cancer Trials Ireland, Galway, Ireland; Department of Surgical Oncology, Breast and Endocrine Surgery Department, Oslo University Hospital, Oslo, Norway; Department of Oncology, Centre for Cancer and Organ Diseases, Rigshospitalet, Copenhagen, Denmark; Department of Clinical Medicine, University of Copenhagen, Copenhagen, Denmark; Department of Oncology, Faculty of Medicine and Health, Örebro University, Örebro, Sweden; Department of Medical Epidemiology and Biostatistics, Karolinska Institutet, Stockholm, Sweden; Europa Donna, ABC Global Alliance, Ljubljana, Slovenia; Department of Breast and Endocrine Surgery, Okayama University Hospital, Okayama, Japan; Faculty of Health and Medical Sciences, University of Copenhagen, Copenhagen, Denmark; Department of Breast Surgery, Gentofte Hospital, Gentofte, Denmark; Department of Hematology, Oncology and Radiation Physics, Skåne University Hospital, Lund, Sweden; Department of Oncology, Faculty of Medicine, Institute of Clinical Sciences Lund, Lund University, Lund, Sweden

## Abstract

**Background:**

Axillary lymph node dissection (ALND) in breast cancer causes substantial arm morbidity and long-term functional impairment. Less extensive axillary surgery, such as targeted axillary dissection (TAD), significantly reduces this risk. Although omission of ALND is standard in clinically node-negative disease with limited sentinel node involvement, this is not the case for patients with clinically node-positive disease undergoing upfront surgery. The aim of the SENOMAC-ULTRA trial is to evaluate whether TAD can safely replace ALND in patients with clinically node-positive breast cancer receiving upfront surgery.

**Methods:**

SENOMAC-ULTRA is a prospective, international, multicentre, randomized non-inferiority trial. Adults with stage II–III invasive breast cancer and axillary metastases detectable by ultrasound and confirmed by fine needle aspiration or core biopsy are eligible for inclusion. Participants are randomized 1 : 1 to TAD (removal of marked metastatic nodes plus sentinel lymph node biopsy) or standard ALND. The primary endpoint is recurrence-free survival, assessed for non-inferiority using a Cox proportional hazards model. Secondary endpoints include overall survival, locoregional recurrence, regional nodal recurrence, distant relapse-free survival, invasive breast cancer-free survival, patient-reported arm morbidity, and health-related quality of life, as well as performance measures of axillary ultrasound and marking techniques. A sample size of 1380 patients has been calculated to provide 81% power to exclude a hazard ratio > 1.47 for recurrence-free survival at 5 years, adopting a non-inferiority margin of 4.5%. Follow-up is planned for 10 years. The trial protocol has been approved by the Swedish Ethical Review Authority (Dnr 2025-07730-01); each participating country will obtain local ethics approval.

**Conclusion:**

It is anticipated that the SENOMAC-ULTRA trial will fill an important knowledge gap guiding the surgical management of patients with clinically node-negative breast cancer. Registration number: NCT06869629 (https://clinicaltrials.gov).

## Teaser

The randomized SENOMAC-ULTRA trial aims to evaluate whether targeted axillary dissection is non-inferior to axillary lymph node dissection in patients with confirmed axillary lymph node metastases who receive upfront surgery. The trial will include 1380 patients in at least 10 countries. The primary endpoint is recurrence-free survival.

## Introduction

Axillary lymph node dissection (ALND) in breast cancer is the major contributor to significant arm morbidity and long-term negative impacts on patient-reported arm function^1–3^. Several prospective randomized trials have shown the non-inferiority of the omission of ALND in patients with clinically node-negative breast cancer who undergo primary surgery and are diagnosed with one to two metastases on sentinel lymph node biopsy (SLNB)^1,3–7^. However, the use of adjuvant radiotherapy (RT) and the eligibility of patients undergoing mastectomy varied among trials. In the SENOMAC trial, which randomly assigned patients with one to two macrometastases on SLNB to either completion ALND or its omission and reported non-inferiority of the de-escalated axillary surgical approach in 2024, 89% of patients in both groups received RT, including nodal target volumes^4,8^. Consequently, current international guidelines do not recommend completion ALND in this patient population^9^, whereas recommendations for adjuvant RT differ^10^.

Patients with axillary metastases detected before surgery have a higher nodal burden than those in whom metastases are identified on SLNB. In a study by Caudle *et al*.^11^, patients with T1–2 node-positive breast cancer, an abnormal axillary ultrasound (AUS), and positive fine needle aspiration cytology had a higher mean number of positive nodes (4.1 *versus* 2.2) and more often a pathological (p) N2–3 status (35% *versus* 11%) on ALND than clinically node-negative (cN0) patients undergoing SLNB before ALND. Still, 52% of patients had only one to two positive nodes overall^11^, a figure questioning the role of ALND. This was corroborated by another study^12^ in which 47% of cN0 patients with an abnormal AUS and a positive preoperative biopsy had only one to two positive nodes on ALND, a figure that increased to 59% for a single abnormal node on AUS.

Current National Comprehensive Cancer Network guidelines^9^ are for SLNB or targeted axillary dissection (TAD) in patients with limited nodal involvement on AUS and negative palpation planned for upfront surgery. Although TAD is a concept otherwise primarily used in the neoadjuvant setting, a similar strategy named tailored axillary surgery (TAS) is currently being tested in the TAXIS trial, which includes node-positive breast cancer both in upfront surgery and after neoadjuvant treatment. TAS is slightly different to TAD, being defined ‘by the sentinel lymph node procedure in combination with the selective removal of all palpable disease and documentation of the removal of the initially biopsy-proven and clipped lymph node metastasis by specimen radiography’^13^. Because intraoperative palpation and removal of obviously abnormal lymph nodes is part of SLNB, differences in nodal yield or precision are expected to be minimal. After TAS confirming node positivity, randomization is between regional nodal RT including the axilla and ALND plus regional nodal RT excluding the dissected axilla^13^. In an early analysis, the proportion of patients undergoing upfront surgery was 67.0% overall, but rates of primary systemic therapy increased significantly over time^14,15^. Another prospective trial addressing surgical de-escalation in clinically node-positive patients in the upfront setting is the announced randomized TADPOLE trial (NCT04671511), which will include patients with limited nodal involvement (one to two suspicious axillary lymph nodes).

The SENOMAC trial was intended^4^ to shed light on the question as to whether ALND could also be omitted in the subgroup of patients with an abnormal AUS and a confirmative preoperative biopsy; however, a negative clinical examination was mandatory. Overall, 347 patients with an abnormal AUS were registered, only 36 of whom had positive preoperative fine needle aspiration cytology. With these low numbers, it is impossible to assess this subgroup separately. Thus, the aims of the SENOMAC-ULTRA trial are to investigate the replacement of ALND with a TAD in clinically node-negative patients with a positive AUS and to broaden the inclusion criteria to clinically node-positive patients with cytological or histopathological confirmation of nodal involvement.

## Methods

SENOMAC-ULTRA is a prospective international multicentre randomized non-inferiority trial.

**Fig. 1:**
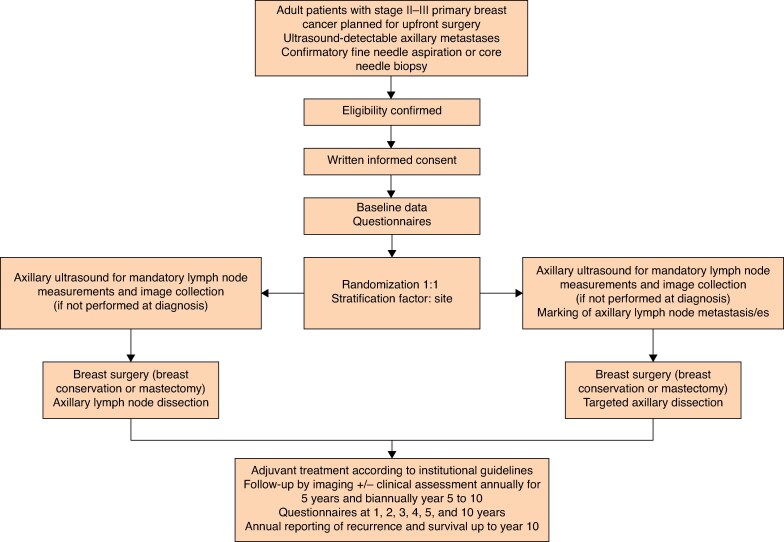
Flow chart for eligibility and enrolment in the SENOMAC-ULTRA trial

Adult patients scheduled for primary surgery for a primary invasive breast cancer clinical stage II–III with AUS-detectable axillary metastases confirmed by preoperative fine needle aspiration or core needle biopsy are eligible for inclusion (*Fig. 1*). Written informed consent is mandatory. Patients with inflammatory or locally advanced breast cancer or disseminated disease are not eligible for inclusion. In accordance with the Eight Edition of the AJCC Cancer Staging Manual^16^, the ‘clinical categorization of cancer is based on findings of history, physical examination, and any imaging studies that are done’. Eligibility criteria are presented in *Table 1*. Further clarification of the eligibility of specific clinical nodal stages is provided in *Table 2*.

**Table 1:** Eligibility criteria

Inclusion criteria	Exclusion criteria
Patients with primary invasive breast cancer clinical stage II–III Palpable or non-palpable axillary metastases visible by ultrasound (other imaging accepted if confirmatory ultrasound is performed) and confirmed by fine needle aspiration or core biopsy Written informed consent Age ≥ 18 years	Distant metastases Inflammatory or locally advanced breast cancer Ipsilateral metastases in internal mammary, infra- or supraclavicular lymph nodes without confirmed axillary nodal involvement Nodes fixed to each other or to neighbouring structures on palpation or imaging History of contralateral invasive breast cancer in the past 5 years Bilateral invasive breast cancer if: (i) any side meets any exclusion criteria; or (ii) both sides meet all inclusion criteria Pregnancy Preoperative systemic treatment (a short course of preoperative endocrine therapy < 3 months is allowed) Medical contraindications for or expressed preoperative wish to refrain from radiotherapy or the recommended adjuvant systemic treatment that complies with standard of care, taking age and co-morbidity into consideration Inability to absorb or understand the meaning of the study information (for example, through disability, inadequate language skills, or dementia)

**Table 2:** Eligibility of clinical nodal staging subcategories

Clinical nodal stage	Definition	Eligible
cN1	Metastasis in movable ipsilateral level I, II axillary lymph nodes	Yes
cN2a	Metastasis in ipsilateral level I, II axillary lymph nodes fixed to one another (matted)	No
cN2b	Metastasis only in clinically detected ipsilateral internal mammary nodes and in the absence of clinically evident level I, II axillary lymph node metastasis	No
cN3a	Metastasis in ipsilateral infraclavicular (level III axillary) lymph nodes with or without level I, II axillary lymph node involvement	Only if with axillary lymph node involvement
cN3b	Metastasis in clinically detected ipsilateral internal mammary lymph nodes and clinically evident axillary lymph nodes	Yes
cN3c	Metastasis in ipsilateral supraclavicular lymph nodes with or without axillary or internal mammary lymph node involvement	Only if with axillary lymph node involvement

Because the accuracy of AUS is highly user dependent, no specific maximum number of suspicious lymph nodes on AUS is applied. In the event of suspected extensive involvement of the axilla on AUS or other preoperative imaging modalities or on palpation during surgery, the performance of ALND is based on clinical judgement with or without intraoperative histopathological confirmation. An intraoperative or postoperative decision to perform ALND in a patient assigned to the intervention group does not lead to exclusion from the trial, but is documented in the trial database.

Criteria for suspicious nodes on AUS may vary between institutions and countries. As a guidance, a cortical thickness > 3 mm or the absence of a fatty hilum are recommended as the main criteria for the identification of metastatic lymph nodes by AUS. Representative still images of the most suspicious nodes should be stored and submitted to the central quality assurance database.

### Intervention

Patients assigned to the intervention group undergo TAD, which implies the removal of previously marked metastatic nodes plus SLNB, which includes the removal of stained and/or radioactive lymph nodes plus nodes that are clearly abnormal on intraoperative palpation. Marking of at least one biopsied nodal metastasis is mandatory and should ideally be performed after randomization. Any routinely used markers, such as clips, guidewires, carbon suspension, magnetic or radioactive seeds, radar reflectors, fluorescence detection, or radiofrequency identification tags, can be used. Marking of more than one node is optional. There is no maximum limit on the number of nodes that can be removed, but it is recommended that not more than four lymph nodes are removed.

In the event of localization failure, SLNB including the removal of clearly suspicious nodes on intraoperative palpation suffices. If multiple nodes have been marked, the identification of at least one is required. If neither the marked lymph node/s nor a sentinel lymph node can be identified during surgery, an ALND or representative sampling should be performed.

### Control

Patients assigned to the control group undergo upfront ALND of anatomical levels 1 and 2 with the aim of removing at least ten lymph nodes. Patients in whom the postoperative histopathology report identifies fewer than ten nodes are not excluded from the trial but are handled in sensitivity analyses.

### Endpoints, variables, and measures

The primary endpoint is recurrence-free survival (RFS) in accordance with STEEP 2.0 criteria^17^. RFS is measured from the date of randomization until the event date of any invasive local, regional, or distant breast cancer recurrence or death from any cause. Contralateral breast cancer is not defined as an event. Patients without any such event are censored at the date of last follow-up. This endpoint will be assessed for non-inferiority. The primary estimand is the hazard ratio of the time to local, regional, or distant breast cancer recurrence or death, comparing patients undergoing upfront TAD with patients undergoing upfront ALND among adults with primary stage II–III breast cancer and AUS-detectable axillary metastases.

Secondary endpoints are as listed below.

1. Overall survival is measured from the date of randomization until the date of death from any cause. Participants who are alive are censored at the date of last follow-up. This endpoint will be assessed for non-inferiority.
2. Locoregional recurrence is measured from the date of randomization until the date of a local or regional recurrence, as defined above. This endpoint will be assessed for non-inferiority.
3. Regional nodal recurrence is measured from the data of randomization until the date of a nodal recurrence in the ipsilateral axilla, supraclavicular, or internal mammary regions. This endpoint will be assessed for non-inferiority.
4. Distant RFS is measured from the date of randomization until the date of any first distant recurrence, as defined above. This endpoint will be assessed for non-inferiority.
5. Invasive breast cancer-free survival is measured from the date of randomization until the date of death from invasive breast cancer recurrence. Participants without a breast cancer recurrence will be censored at the date of death from other causes or the date of last follow-up, if still alive. This endpoint will be assessed for non-inferiority.
6. Patient-reported arm morbidity will be assessed using the Lymphedema Functioning, Disability and Health questionnaire (Lymph-ICF), completed by study participants at baseline, annually for 5 years, and again after 10 years. This endpoint will be assessed for superiority at multiple time points (for example 1, 3, 5, and 10 years after surgery), including longitudinal analyses.
7. Health-related quality of life will be assessed using the European Organisation for Research and Treatment of Cancer (EORTC) Core Quality of Life (QLQ-30) and Breast Cancer (BR-42) questionnaires completed by study participants at baseline, annually for 5 years, and again after 10 years. In addition, sense of coherence will be assessed using the SOC-13 questionnaire at baseline to allow adjustment of health-related quality of life outcomes for individual coping capabilities in challenging life situations. This endpoint will be assessed for superiority at multiple time points (for example 1, 3, 5, and 10 years after surgery), including longitudinal analyses.
8. Performance measures of AUS will be evaluated documenting cortical thickness (millimetres), long- and short-axis diameter, the presence of a focal cortical bulge of > 3 mm, partial or complete effacement of the fatty hilum, and non-hilar cortical blood flow on colour Doppler imaging. The positive predictive value, sensitivity, and specificity of single and combined features for high nodal burden (four or more nodal metastases) will be evaluated. This endpoint will be assessed separately in the ALND group because ALND is necessary for determining high nodal burden.
9. Performance measures of different markers in TAD will be evaluated by comparing the successful removal of the inserted marker between different marking strategies. This endpoint will be compared between different groups of marking strategies (for example, specific markers as well as visually guided identification *versus* probe-guided identification).

Further aims of SENOMAC-ULTRA are as follows:

- evaluation of the SENOMAC nomogram^18^ predicting high nodal burden in patients with one to two sentinel lymph node macrometastases and its potential application to the SENOMAC-ULTRA population (a revised nomogram for high nodal burden will be created if necessary, incorporating AUS features readily available in routine care if proven to improve the nomogram’s predictive accuracy)
- assessment of the potential impact of axillary surgery on adjuvant systemic treatment (that is, both indications and the specific regimens)
- assessment of health economy by the EORTC QLQ-30 and EQ-5D-5L™ questionnaires annually for 5 years and again after 10 years.

### Randomization

Patients will be randomized 1:1 to either upfront ALND (control) or TAD (intervention). Randomization lists will be prepared using randomly permuted blocks of sizes 6 and 8 and will be stratified by site. Further stratification factors, such as type of breast surgery, received RT, and subtype, were considered but will instead be handled in sensitivity analyses. The randomization lists will be produced using the statistical software package Stata^®^ (StataCorp. 2023. Stata Statistical Software: Release 18. College Station, TX, USA: StataCorp LLC), and will then be uploaded into the REDCap electronic case report form system. Personnel enrolling participants and those who assigning them to their respective groups will not have access to the random allocation sequence. This is not a blinded trial, and it will not be possible to mask treatment assignment from trial personnel. The trial statistician will be blinded to participants’ randomized allocation until the target number of events has been observed and the final data set is downloaded.

### Adjuvant treatment

Systemic adjuvant treatment is to be administered in accordance with national or institutional guidelines.

RT follows national or institutional guidelines, as detailed in *Table 3*. Target volumes must be delineated as separate clinical target volumes according to European Society of Radiotherapy and Oncology guidelines^19^. Dose, fractionation, and treatment time must be used in accordance with relevant national or institutional guidelines. The recommended dose in locoregional RT is 40 Gy in 15 fractions given 5 days/week. Details on RT must be reported prospectively in the trial database. A comprehensive quality assurance of RT will be performed to underpin the conclusions of the trial endpoints. For this purpose, sites are encouraged to continuously submit each participant’s treatment plan for central review. As a minimum, the first five RT plans should be submitted; thereafter, every second to third plan should be submitted in the order of enrolment.

**Table 3:** Radiotherapy summary

Whole breast radiotherapy	Mandatory after breast-conserving surgery (that is, partial breast radiotherapy is not accepted)
Chest wall irradiation after mastectomy	According to institutional guidelines
Regional lymph node radiotherapy	According to institutional guidelines
Tumour bed boost	According to institutional guidelines
Volume definition	The remaining breast/chest wall and each lymph node level must be delineated as separate volumes according to ESTRO guidelines
Organs at risk	The heart, ipsilateral humeral head, ipsilateral lung, and spinal canal must be delineated for all patients
Reporting/quality assurance	Radiotherapy details reported to the trial database for all patients; radiotherapy plans submitted for central review

ESTRO, European Society of Radiotherapy and Oncology.

### Monitoring and follow-up

Each patient is to be followed up for 10 years after surgery in accordance with national and/or regional follow-up schedules. The minimum follow-up includes annual imaging (mammography, ultrasound and/or magnetic resonance imaging), with or without clinical examination, for 5 years and then imaging every 2 years, with or without clinical examination or telephone contact. Telephone contact at 5 years is mandatory. However, data on ongoing treatment, recurrence, and survival must be completed annually until 10 years after enrolment, even if the patient has not been to a clinical examination or imaging follow-up.

### Estimated sample size and power

The aim of this trial is to show that the intervention is non-inferior to the standard of care regarding the primary endpoint of RFS. In the SENOMAC trial, patients in whom ALND was omitted had an estimated 5-year RFS of 89.7%^4^, which is comparable to recently reported rates in similar trials such as in the two randomization groups of the INSEMA trial’s second randomization^20^ (invasive disease-free survival 86.6% and 93.8%, respectively) and of the SINODAR-ONE trial (RFS 96.4% and 95.6%, respectively)^7^. The higher clinical risk in SENOMAC-ULTRA, including patients with higher nodal stages than SENOMAC, should be balanced by the fact that CDK4/6 inhibitors have become the standard of care for patients with oestrogen receptor-positive breast cancer^21^, who are expected to be the predominant population in SENOMAC-ULTRA, just as in SENOMAC (93.4%)^4^.

Thus, assuming a 5-year RFS rate of 89.7% in both groups, the study aims to show that the 5-year RFS is non-inferior in the intervention arm using a non-inferiority margin of 4.5% (that is, 5-year RFS of 85.2%). The non-inferiority margin was selected to be lower than the non-inferiority margin in similar trials (for example, 5% in the TAXIS trial^13^ and 6% in the Alliance A011202 trial^22^). Based on this, the study needs to show that the upper limit of the 90% confidence interval (equivalent to a one-sided alpha of 0.05) of the hazard ratio is below 1.47. Using the method described by Jung *et al*.^23^ gives a sample size of 1230, recruited equally over 5 years and a total trial duration of 9 years, with 162 events required for 80% power. However, because recruitment is expected to be slower in the first 2 years, a simulation was used to investigate the effect of this on the required sample size. Assuming exponential survival times and non-proportional recruitment over time, the total sample size increased to 1380 and achieved power increased to 81%. Simulation was performed in Stata^®^ (StataCorp. 2023. Stata Statistical Software: Release 18. College Station, TX, USA: StataCorp LLC), using 10 000 simulated data sets.

### Statistical analysis

The primary endpoint of RFS will be used to estimate the hazard ratio between the two trial groups using a Cox proportional hazards regression model. Non-inferiority will be declared if the upper bound of the 90% confidence interval of the estimate of the treatment effect is lower than 1.47. The model will adjust for strata used in the randomization procedure (site); no other preplanned covariate adjustment will be performed. If there is strong evidence of non-proportional hazards for the treatment parameter, a restricted mean survival time at 5 years after randomization will be used to estimate the effect of the treatment and an absolute inferiority margin of 4.5% will be used.

Analysis will be performed in both the intention-to-treat and per-protocol populations, with equal weight given to each result, following current European Medicines Agency guidelines^24^. If European Medicines Agency guidance changes while the trial is underway, consideration will be given to adapting the statistical approach to follow best practice and the guidelines in place at the time the final analysis is performed.

Secondary endpoints will be assessed according to the same principles as the primary endpoint. All non-inferiority endpoints will be assessed using the lower or upper (as appropriate) bound of a 90% confidence interval. All superiority endpoints will be assessed using a two-sided alpha of 0.05. More details will be provided in the statistical analysis plan, which will be written, approved, and made publicly available before the trial statistician accesses unblinded data.

### Ethics and dissemination

The trial protocol has been approved by the Swedish Ethical Review Authority (Dnr 2025-07730-01). Each participating country will obtain their own ethics permissions based on the currently valid trial documents.

ALND is routinely performed without preoperative axillary marking procedures. For participants randomized to the intervention group, the marking procedure may result in discomfort and potentially temporary pain, a haematoma, or, rarely, an infection. In addition, a degree of increased risk for axillary recurrence among individual patients who do not undergo ALND cannot be ruled out. However, studies in which patients received systemic treatment according to modern standards for lymph node-positive patients showed no impact of reduced extent of axillary surgery on survival or risk of recurrence, including axillary recurrences^1,3,4,7^. In addition, this potential risk increase must be weighed against the significant benefit of less arm morbidity associated with less extensive surgery. ALND is currently increasingly abandoned in diverse patient populations, and there is a risk that ALND omission will be implemented in routine care or individual practice before sufficient evidence is available, which is why it is crucial to explore this approach in a trial.

Publications of the primary and secondary endpoints are collaborative efforts by steering committee members, statisticians, advisors, the RT quality assurance team, central trial coordinator, and patient representatives, who are all included as members of The SENOMAC-ULTRA Trialists Group.

## Data Availability

Deidentified participant data may be made available upon reasonable request to the coordinating investigator if accompanied by all relevant ethical and legal permissions. The earliest date of data sharing is 3 months after the publication of the primary endpoint. Any request for data will be discussed in the international steering committee, and only requests that fulfil all necessary requirements will be accepted.
